# Supplementary material for: Cooperation-based concept formation in male bottlenose dolphins
Source: Nat Commun. 2021 Apr 22;12:2373. doi: 10.1038/s41467-021-22668-1 (PMC8062458; doi:10.1038/s41467-021-22668-1)
Supplement: Supplementary file 1 — Supplementary Information [file 41467_2021_22668_MOESM1_ESM.pdf]

Cooperation-based concept formation in male bottlenose dolphins

Stephanie L. King[1,2*], Richard C. Connor[3], Michael Krützen[4] and Simon J. Allen[1,2,4]

[1] School of Biological Sciences, University of Bristol, Bristol, BS8 1TQ, United Kingdom

[2] School of Biological Sciences, University of Western Australia, Crawley, WA, 6009, Australia

[3] Biology Department, University of Massachusetts Dartmouth, North Dartmouth, MA 02747, USA

[4] Evolutionary Genetics Group, Department of Anthropology, University of Zurich, CH-8057 Zurich, Switzerland

* Corresponding Author: Stephanie King, School of Biological Sciences, University of Bristol, Bristol, BS8 1TQ, UK; stephanie.king@bristol.ac.uk; ORCID: 0000-0003-2293-9185

Supplementary Information

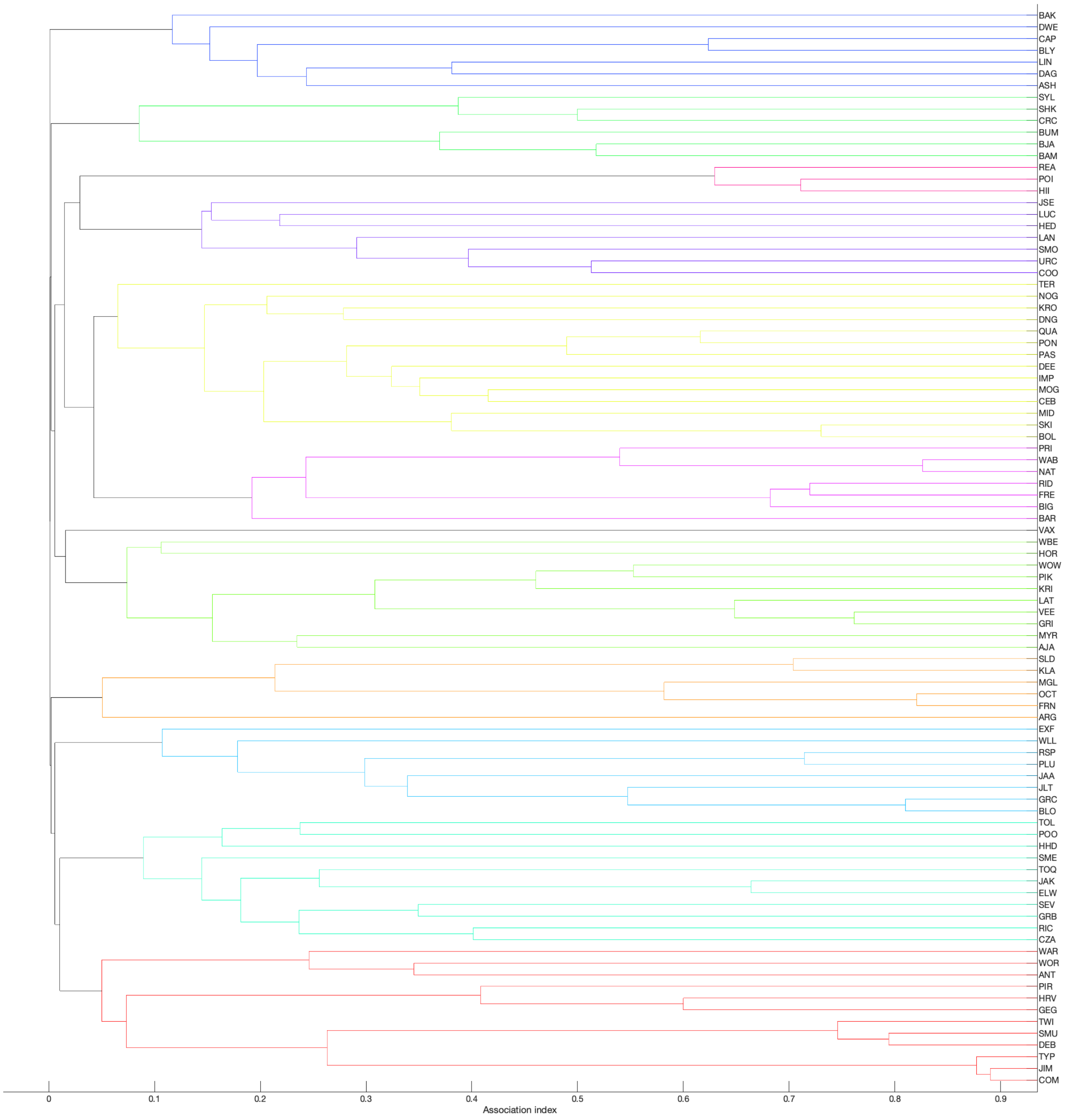

Supplementary Figure 1. Average-linkage cluster diagram, based on association indices (Simple Ratio Index) among 92 male bottlenose dolphins with extensive home range overlap from 2001 to 2019, illustrating the range of alliance relationships in Shark Bay. The RR alliance (JES, LUC, HED, LAN, SMO, URC, COO) is shown in dark purple, KS alliance (TER, NOG, KRO, DNG, QUA, PON, PAS, DEE, IMP, MOG, CEB, MID, SKI, BOL) in yellow and the PD alliance (PRI, WAB, NAT, RID, FRE, BIG, BAR) in light purple.

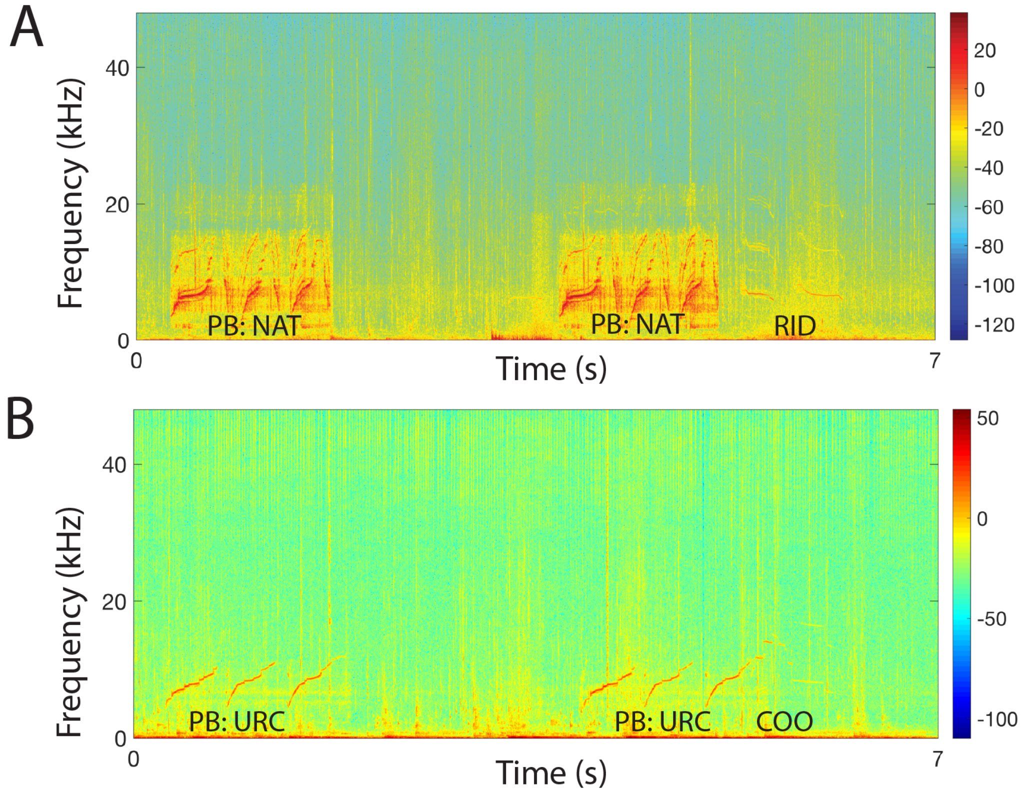

Supplementary Figure 2. Vocal response to alliance playbacks. **(A)** Spectrogram (sampling rate: 96 000 Hz, FFT length: 1024) showing vocal response by RID to playback (PB) of second-order ally NAT, with whom he has never been documented partnering as a first-order ally and shared a bond strength (SRI) of only 0.19. **(B)** Spectrogram showing vocal response of COO to PB of second-order ally URC, with whom his bond strength (SRI) was 0.78. COO was foraging and showed no observable behavioural response but produced his own signature whistle in response to the second URC whistle in this PB. Amplitude intensity (dB) shown in the colour bar. Spectrograms of all males in this study are shown in Fig. 1 in King et al. [1].

1. King, S. L. *et al.* Bottlenose dolphins retain individual vocal labels in multi-level alliances. *Curr. Biol.* **28**, 1993–1999 (2018).

Influence of group size on playback response

The majority of playbacks were conducted to males when they were on their own, with smaller proportions in pairs or trios either with or without a female. For groups, we always measured the response of the male that showed the strongest response, which was always the male that responded first. However, to ensure that the presence of group members was not influencing the strength of response given to the playback treatments, we compared response strengths (response duration and approach distance) for individuals on their own and individuals in groups. We compared responses to second- and third-order alliance playbacks separately to ensure the presence of group members was not influencing the response of individuals to one playback treatment and not the other. We detected no difference in response strength between individuals on their own at the time of playback and individuals in groups for either playback type or response measure:

Response duration

Individual versus group for second-order alliance playbacks (Welch's t test (two-sided), $t = -1.32$, $p = 0.2$)

Individual versus group for third-order alliance playbacks (Welch's t test (two-sided), $t = -0.01$, $p = 0.9$)

Approach distance

Individual versus group for second-order alliance playbacks (Welch's t test (two-sided), $t = -0.98$, $p = 0.3$)

Individual versus group for third-order alliance playbacks (Welch's t test (two-sided), $t = 0.60$, $p = 0.5$)

Supplementary Table 1. Results of linear mixed-effect full-null model comparison. Support to reject the null model using Chi-squared test.

		Model	Deviance	Pr(>Chi)
**Model 1**	Full	lmer (response duration ~ PB type + consort + SRI + PB order + (1|subject ID) + (1| caller ID)	3.43.13	< 0.0001
	Null	lmer (response duration ~ consort + SRI + PB order + (1|subject ID) + (1| caller ID)	360.04	
**Model 2**	Full	lmer (approach distance ~ PB type + consort + SRI + PB order + (1|subject ID) + (1| caller ID)	288.76	0.001
	Null	lmer (approach distance ~ consort + SRI + PB order + (1|subject ID) + (1| caller ID)	299.09	
**Model 3**	Full subset (w/out RR)	lmer (response duration ~ PB type + consort + SRI + PB order + (1|subject ID) + (1| caller ID)	232.70	0.002
	Null subset (w/out RR)	lmer (response duration ~ consort + SRI + PB order + (1|subject ID) + (1| caller ID)	241.79	
**Model 4**	Full subset (w/out RR)	lmer (approach distance ~ PB type + consort + SRI + PB order + (1|subject ID) + (1| caller ID)	201.71	0.003
	Null subset (w/out RR)	lmer (approach distance ~ consort + SRI + PB order + (1|subject ID) + (1| caller ID)	210.24	
**Model 5**	Full	glmer (orient to source ~ PB type + consort + SRI + PB order + (1|subject ID) + (1| caller ID)	37.31	0.02
	Null	glmer (orient to source ~ consort + SRI + PB order + (1|subject ID) + (1| caller ID)	42.24	
