# Supplementary material for: Cooperation-based concept formation in male bottlenose dolphins
Source: Nat Commun. 2021 Apr 22;12:2373. doi: 10.1038/s41467-021-22668-1 (PMC8062458; doi:10.1038/s41467-021-22668-1)
Supplement: Supplementary file 3 — Reporting Summary [file 41467_2021_22668_MOESM3_ESM.pdf]

nature research

Corresponding author(s): Stephanie King

Last updated by author(s): Aug 22, 2020

Reporting Summary

Nature Research wishes to improve the reproducibility of the work that we publish. This form provides structure for consistency and transparency in reporting. For further information on Nature Research policies, see our Editorial Policies and the Editorial Policy Checklist.

Statistics

For all statistical analyses, confirm that the following items are present in the figure legend, table legend, main text, or Methods section.

n/a	Confirmed	
☐	☒	The exact sample size (*n*) for each experimental group/condition, given as a discrete number and unit of measurement
☐	☒	A statement on whether measurements were taken from distinct samples or whether the same sample was measured repeatedly
☐	☒	The statistical test(s) used AND whether they are one- or two-sided<br>*Only common tests should be described solely by name; describe more complex techniques in the Methods section.*
☐	☒	A description of all covariates tested
☐	☒	A description of any assumptions or corrections, such as tests of normality and adjustment for multiple comparisons
☐	☒	A full description of the statistical parameters including central tendency (e.g. means) or other basic estimates (e.g. regression coefficient) AND variation (e.g. standard deviation) or associated estimates of uncertainty (e.g. confidence intervals)
☐	☒	For null hypothesis testing, the test statistic (e.g. *F*, *t*, *r*) with confidence intervals, effect sizes, degrees of freedom and *P* value noted<br>*Give P values as exact values whenever suitable.*
☒	☐	For Bayesian analysis, information on the choice of priors and Markov chain Monte Carlo settings
☒	☐	For hierarchical and complex designs, identification of the appropriate level for tests and full reporting of outcomes
☒	☐	Estimates of effect sizes (e.g. Cohen's *d*, Pearson's *r*), indicating how they were calculated

Our web collection on statistics for biologists contains articles on many of the points above.

Software and code

Policy information about availability of computer code

Data collection	No software were used in the data collection
Data analysis	All statistical procedures were conducted in R 3.6.1 (R project for statistical computing; GNU project). We used R packages irr, lme4, car, influence.ME, and asnipe. Localization accuracy of the array was calculated using custom-written MATLAB (2018b) routines (see Quick et al 2008 as referenced in the manuscript). We used Adobe Audition (version 12.1.0) to create playback stimuli and visualise acoustic data. We used QuickTime player (v10.5) to analyse videos.

For manuscripts utilizing custom algorithms or software that are central to the research but not yet described in published literature, software must be made available to editors and reviewers. We strongly encourage code deposition in a community repository (e.g. GitHub). See the Nature Research guidelines for submitting code & software for further information.

Data

Policy information about availability of data

All manuscripts must include a data availability statement. This statement should provide the following information, where applicable:

- Accession codes, unique identifiers, or web links for publicly available datasets
- A list of figures that have associated raw data
- A description of any restrictions on data availability

The data that support the findings of this study will be deposited in the Dryad Digital Repository (doi:10.5061/dryad.rbnzs7hb4). Source Data for the linear mixed model analyses and data presented in Fig 3 have been provided in the Source Data file.

Field-specific reporting

Please select the one below that is the best fit for your research. If you are not sure, read the appropriate sections before making your selection.

☒ Life sciences ☐ Behavioural & social sciences ☐ Ecological, evolutionary & environmental sciences

For a reference copy of the document with all sections, see nature.com/documents/nr-reporting-summary-flat.pdf

Life sciences study design

All studies must disclose on these points even when the disclosure is negative.

Sample size	Sample sizes for the sound playback experiments were determined by the number of male dolphins whose identity call had been identified. We have three complete alliances comprising 14 males (independent samples). Our minimum number of independent samples for a playback study is 12, as this is the minimum number required to find a statistical effect if the effect is small (see references below). However, our number of playback experiments (repeated measures design) far exceeded both these papers at 40.<br><br>King SL & Janik VM (2013). Bottlenose dolphins can use learned vocal labels to address each other. Proceedings of the National Academy of Sciences USA 110 (32): 13216-13221. (n=12)<br><br>Janik VM, Sayigh LS, Wells RS (2006) Signature whistle shape conveys identity information to bottlenose dolphins. Proceedings of the National Academy of Sciences USA 103(21):8293–8297. (n=14)
Data exclusions	No data were excluded from the analysis.
Replication	Some playback experiments were repeated (with different stimuli) to the same males and the ID of these males were included as a random effect in our model: we conducted 40 playbacks to 14 males, where each male was subject to a mean of 4.7 playbacks (range 1-8). We also ran a repeated analysis where we removed a subset of data (as described in the paper) and the same results were confirmed. We haven't replicated this experiment with a different group of animals.
Randomization	We randomised playback treatment so that the order of playback stimuli was randomised for each male to ensure there were no order effects in the behavioral response.
Blinding	One observer coded all 40 playback videos from the UAV footage, as well as the time-synchronized audio recordings. A second observer, blind to playback treatments and the first observer's scores, independently coded variables. We then conducted an inter-observer reliability analysis using the intraclass correlation coefficient (ICC) for two-way models in the irr package in R 3.6.1 (R project for statistical computing; GNU project), and found strong agreement between the two observers (ICC = 0.976, P = < 0.0001, CI = 0.967 - 0.983).

Reporting for specific materials, systems and methods

We require information from authors about some types of materials, experimental systems and methods used in many studies. Here, indicate whether each material, system or method listed is relevant to your study. If you are not sure if a list item applies to your research, read the appropriate section before selecting a response.

Materials & experimental systems

n/a	Involved in the study	
☒	☐	Antibodies
☒	☐	Eukaryotic cell lines
☒	☐	Palaeontology and archaeology
☐	☒	Animals and other organisms
☒	☐	Human research participants
☒	☐	Clinical data
☒	☐	Dual use research of concern

Methods

n/a	Involved in the study	
☒	☐	ChIP-seq
☒	☐	Flow cytometry
☒	☐	MRI-based neuroimaging

Animals and other organisms

Policy information about studies involving animals; ARRIVE guidelines recommended for reporting animal research

Laboratory animals	The study did not involve laboratory animals
Wild animals	We worked with free-ranging adult male bottlenose dolphins (Tursiops aduncus) in the eastern gulf of Shark Bay, Western Australia, where our long-term dolphin research project has been running on a seasonal basis (typically austral winter and spring) since 1982. Males ranged from approximately 28 to 40 years old. Permits for the scientific use of animals were obtained from the Department of Biodiversity, Conservation and Attractions (DBCA), Western Australia.

Field-collected samples	The study did not involve samples collected from the field.
Ethics oversight	The University of Bristol (Animal Welfare and Ethics Review Body) and University of Western Australia (Animal Ethics Committee) granted animal ethics approvals.

Note that full information on the approval of the study protocol must also be provided in the manuscript.
